# Overexpression of Small Heat Shock Protein LimHSP16.45 in *Arabidopsis* Enhances Tolerance to Abiotic Stresses

**DOI:** 10.1371/journal.pone.0082264

**Published:** 2013-12-13

**Authors:** Changjun Mu, Shijia Zhang, Guanzhong Yu, Ni Chen, Xiaofeng Li, Heng Liu

**Affiliations:** Institute of Cell Biology, School of Life Sciences, Lanzhou University, Lanzhou, P.R. China; Arizona State University, United States of America

## Abstract

Small heat shock proteins (smHSPs) play important and extensive roles in plant defenses against abiotic stresses. We cloned a gene for a smHSP from the David Lily (*Lilium davidii* (E. H. Wilson) Raffill var. Willmottiae), which we named *LimHSP16.45* based on its protein molecular weight. Its expression was induced by many kinds of abiotic stresses in both the lily and transgenic plants of *Arabidopsis.* Heterologous expression enhanced cell viability of the latter under high temperatures, high salt, and oxidative stress, and heat shock granules (HSGs) formed under heat or salinity treatment. Assays of enzymes showed that *LimHSP16.45* overexpression was related to greater activity by superoxide dismutase and catalase in transgenic lines. Therefore, we conclude that heterologous expression can protect plants against abiotic stresses by preventing irreversible protein aggregation, and by scavenging cellular reactive oxygen species.

## Introduction

Environmental degradation and abiotic stresses are limiting factors in food production and sustainability. They have become the most severe challenges in agriculture. In response to high temperatures and other abiotic stresses, all organisms universally respond by accumulating heat shock proteins (HSPs) [1]. These HSPs are mainly involved in peptide-folding, protein assembly and transport, protection against irreversible protein denaturation, maintaining the protein in a normal folding state, promoting the degradation of mis-folded proteins under various stresses, and they all can be summed as having “chaperone-like” activities [2]–[6]. At least five types occur in higher plants – the Hsp70 (DnaK) family, chaperonins (GroEL and Hsp60), Hsp90 family, Hsp100 (Clp) family, and small HSP (smHSP) family (12–40 kD). The smHSPs, the most abundant stress-induced proteins, are highly conserved in their C-terminal, where an α-crystallin domain (ACD) is found [1], [7]. Plants contain a wide array of smHSPs that are divided into six classes based on their sequence alignments and immunological cross-reactivity. Three of these classes (CI, CII, and CIII) are located in the cytoplasm or nucleus [8]. The others exist in the endoplasmic reticulum [9], mitochondria and plastids [10].

Functioning as molecular chaperones *in* *vitro* and *in vivo*, smHSPs can prevent irreversible protein aggregation and maintain denatured proteins in a folding-competent state under abiotic stress conditions [11]–[16]. In the absence of such stresses, however, smHSPs can also be produced specifically in reproductive organs at certain developmental stages, including seed maturation and germination, pollen development, and fruit maturation [17]–[18], [4]. Three smHSPs – OsHSP17.0, OsHSP26.7, and OsHSP24.1– are predominantly expressed in the spikes and/or imbibed seed embryos, indicating certain roles for them in pollen development and seed germination [19]. Therefore, many smHSPs can function in either the presence or absence of abiotic stress. For example, AtHSP17.6, an *Arabidopsis* cytoplasmic smHSP, is expressed in heat-shocked leaves but not in untreated control leaves of *Arabidopsis*. However, *Arabidopsis* embryos show high constitutive expression of *AtHSP17.6* in meristematic and pro-vascular tissues [20].

Research on smHSPs has generally emphasized plant tolerances to various environmental stresses, e.g., high temperature [21], salt [22], osmotic pressure [13], and oxidation [23]. Other studies have used genetic modifications to focus on transgenic plants that over-express smHSPs, leading to improved agronomic traits with respect to basal thermotolerance [24]–[25], seed longevity [26], and tolerances to osmotic stress [13] and chilling [27].

*Lilium davidii* (E. H. Wilson) Raffill var. Willmottiae (David Lily) is a perennial herb with high nutritional quality. In China, it is renowned as a good source of food and medicine. Global temperature changes and soil salinization have greatly influenced its cultivation and seriously reduced its productivity. We previously cloned a gene for smHSPs from David Lily, and named it *LimHSP16.45*, based on its protein molecular weight. LimHSP16.45 shares very high identity with other plant cytoplasmic II smHSP, including those from *Arabidopsis*, *Oryza sativa*, *Pisum sativum*, and *Vitis vinifera* [16]. Those earlier studies demonstrated that LimHSP16.45 can act as a protein chaperone, protecting pollen mother cells and tapetal cells against extreme temperatures [16], [28]. To examine further its functioning and molecular mechanism under abiotic stresses, we transformed *LimHSP16.45-GFP* into *Arabidopsis* as our model system. Our goal here was to investigate how its overexpression influences plant tolerance to stress and to improve our overall understanding of the roles played by smHSPs during the stress response.

## Materials and Methods

### Ethics Statement

In our research, David Lily was planted in field of Lanzhou University, and we have got permission of Lanzhou University for our study.

### Total RNA Isolation, Quantitative Real-time PCR, and Statistical Analysis of David Lily

Floral buds were collected from David Lily in the late zygotene to pachytene stages (12–13 mm). Plants had been exposed for 4 h to 4°C or 45°C (low/high temperature stress) or else treated for 7 d with high salt (100 mM NaCl), oxidative stress (1 mM H_2_O_2_), or osmotic stress (250 mM mannitol). Their total RNA was then isolated from the anthers with Trizol Reagent. After treatment with RNase-free DNase I (TaKaRa, Dalian, China) according to the manufacturer’s instructions, 0.5 to 1.0 µg of total RNA was used for qRT-PCR (forward primer 5′-GGATTCGAAGTTCGAAGTG-3′ and reverse primer 5′-ATCTCAATGGCCTTTGGCTC-3′). Assays were performed in triplicate, and the data were expressed as means ± standard errors.

### Generation of Constructs and Transformation of *Arabidopsis*

The cauliflower mosaic virus 35S promoter, plus cDNA of *LimHSP16.45* and a *GFP* sequence were inserted into the pBI101.2 binary vector to produce *35S::LimHSP16.45-GFP* fusions. The construct was introduced into *Agrobacterium tumefaciens* strain GV3101 and subsequently transformed into wild-type (WT) *Arabidopsis* (‘Col-0’) plants by the floral-dip method [29]. As the control, an empty vector was inserted into WT *Arabidopsis*. Expression was then monitored in the leaves, roots, stems, and anthers.

### *Arabidopsis* Growing Conditions, Stress Treatments and Biophysical Analysis of Transgenic *Arabidopsis*

*Arabidopsis* seedlings were grown on a full-strength Murashige and Skoog [30] (MS; pH 5.8) that was supplemented with 1% (w/v) Suc and 1×Gamborg’s vitamins. Seeds were first surface-sterilized with a 20% (v/v) bleach solution, then washed thoroughly with sterile water and placed on MS plates solidified with 1.0% agar. After incubation at 4°C for 2 d in darkness, the seedlings were oriented vertically for growth under a 16-h photoperiod at 22°C. To evaluate the stress tolerance of transgenic *Arabidopsis* that constitutively expresses *LimHSP16.45-GFP* during germination, we placed approximately 50 surface-sterilized seeds each from WT plants and transgenics (T3 generation) on triplicate plates. The MS media was supplemented with or without NaCl, mannitol, or H_2_O_2_. The seeds were incubated at 4°C for 2 d before being placed at 22°C under a 16-h photoperiod. Germination rates or the extent of root elongation were scored every day for 7 to 14 d. To induce high-temperature stress, we incubated approximately 50 surface-sterilized seeds each from WT or transgenic (T3) plants at 4°C for 2 d, then exposed them to 45°C for 1 to 2 h before placing them on an MS medium. Germination rates were scored every day for 7 d, and each experiment was performed at least three times.

### RNA Preparation and RT-PCR of *Arabidopsis*

Total RNA was obtained from 2-week-old seedling tissues, using an RNA isolation kit (TaKaRa). Samples of each tissue type (1 µg) were digested with RNase-free DNase I (TaKaRa) for reverse transcription (RT) with M-MLV reverse transcriptase (Invitrogen, CA, USA). After a 1∶10 dilution was made, 1 µL of the synthesized cDNA was used for RT-PCR. For analysis expression profiles of *LimHSP16.45-GFP*, forward primer is LimHSP16.45-specific, and reverse primer is GFP-specific (forward primer 5′-GGATTCGAAGTTCGAAGTG-3′, and reverse primer 5′-CCATGCCATGTGTAATCCCA-3′). Conditions included 30 cycles of 94°C for 1 min, 60°C for 1 min, and 72°C for 1.5 min; followed by a final 72°C for 5 min. Afterward, gels were run to assay for expression by each independent transgenic line, and 18S rRNA was run as control.

### Microscopy and Image Analysis

Fluorescent specimens were observed with a confocal microscope (Zeiss LSM 510 META laser-scanning fluorescence microscope) equipped with an epifluorescence UV light filter set. To detect GFP, we used a 488-nm excitation level and a BP 505–530 filter.

### Preparation of Crude Extract and Assays of Enzymatic Activity

Fresh leaves (0.2 g) from *Arabidopsis* were ground to fine powder in liquid nitrogen, and suspended in cold 0.2 M phosphate buffer (pH 8.0) containing 1 mM dithiotreitol and 5 mM ethylenediaminetetra-acetic acid (EDTA). After the lysate was centrifuged (16,000×*g*, 15 min, 4°C), the supernatant was recovered and kept on ice. The assay for superoxide dismutase (SOD) activity was performed according to methods of Beyer and Fridovich [31]. Using a 3-mL reaction mixture composed of 50 mM phosphate buffer (pH 7.0), 0.1 mM EDTA, 13 mM methionine, 75 mM NBT, and 50 µL of lysate, we added 2 mM lactochrome to start the reaction. Absorbance was measured at 560 nm. Activity by ascorbate peroxidase (APX) was determined following the H_2_O_2_-dependent oxidation of ascorbic acid (ASC) at 290 nm in a 3-mL reaction mixture comprising 0.3 mM ASC, 0.1 mM H_2_O_2_, 50 mM phosphate buffer (pH 7.8), and 50 µL of lysate. The assay for catalase (CAT) activity followed the protocol of Beaumont et al. [32], which monitored the dismutation of H_2_O_2_ at 240 nm in a 3-mL reaction mixture containing 50 mM phosphate buffer (pH 7.0), 10 mM H_2_O_2_, and 50 µL of lysate.

## Results

### Expression of LimHSP16.45 is Induced by Various Abiotic Stresses in David Lily

**Figure 1:**
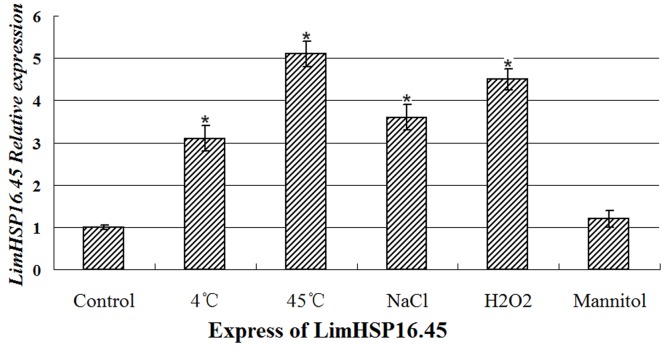
*LimHSP16.45* expression profiles for David Lily under abiotic stresses. qRT-PCR analysis of anthers after 4 h at 4°C or 45°C, or after exposure for 7 d to 100 mM NaCl, 1 mM H_2_O_2_, or 250 mM mannitol. *Indicates significant groups, compared with the control (p<0.05).

Expression of smHSPs can be induced by heat, cold, or other abiotic stresses. Our previous study showed that LimHSP16.45 protects pollen mother cells and tapetal cells against extreme temperatures during the late zygotene to pachytene stages of meiotic prophase I [16], [28]. Therefore, to investigate the relationship between expression and stress, we performed qRT-PCR with anther tissues from David Lily during those developmental stages. Treatments included 4 h at either 4°C or 45°C, or 7 d of exposure to 100 mM NaCl, 1 mM H_2_O_2_, or 250 mM mannitol. Expression was significantly increased in response to either temperature extreme, as well as high salt and oxidative stresses (Fig. 1). However, no significant difference in expression was seen after osmotic stress. These results indicated that LimHSP16.45 has roles in plant responses to multiple abiotic stresses.

### LimHSP16.45-GFP is Constitutively Expressed in Transgenic *Arabidopsis*, and is Localized to the Cell Membrane and Endomembrane System

**Figure 2:**
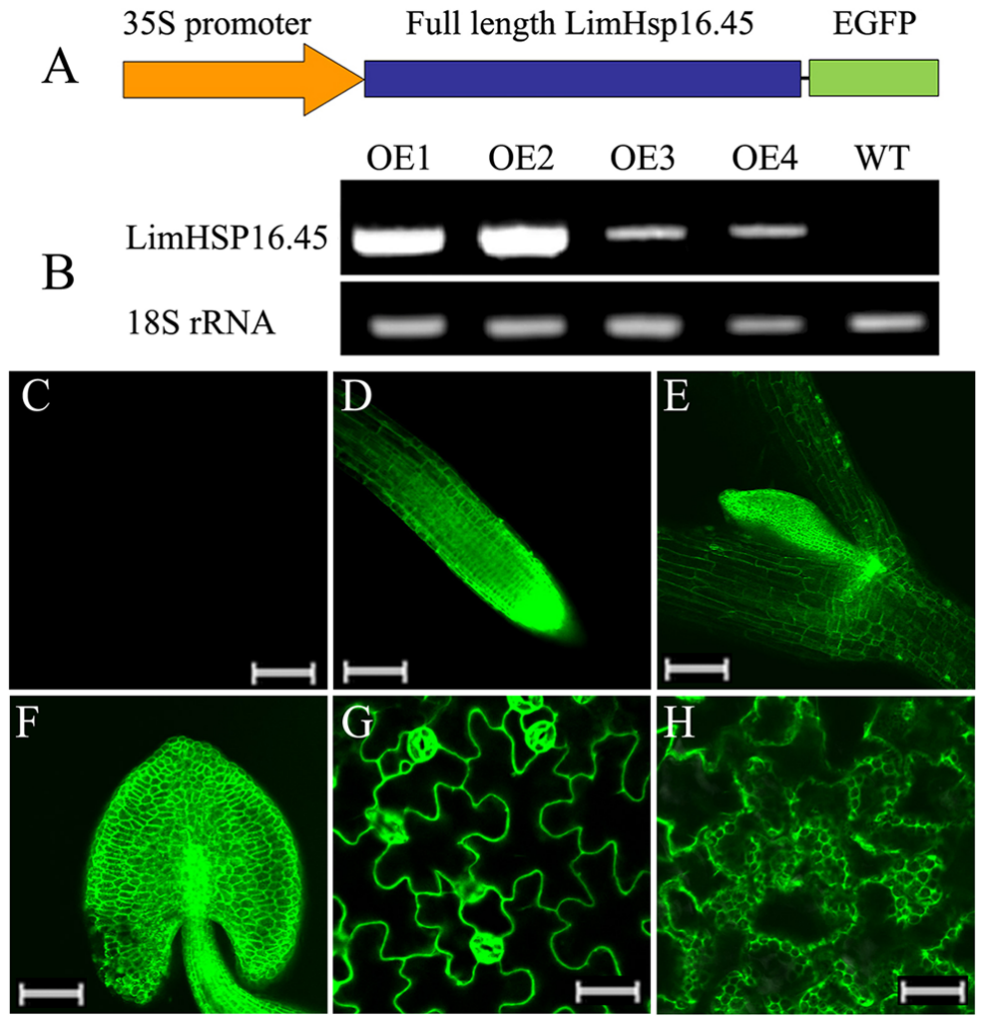
Vector construction and localization of over-expressed *LimHSP16.45-GFP* in *Arabidopsis*. A, Structure of over-expressed LimHSP16.45-GFP fusion protein. B, RT-PCR assay of *LimHSP16.45-GFP* in over-expressing *Arabidopsis*. cDNA was amplified from WT plants and transgenic Lines OE1, 2, 3, and 4. 18S rRNA was loaded as control. *LimHSP16.45-GFP* was highly expressed in apical meristems of roots (D), stems (E), and anthers (F). Protein was localized to membrane (G) and endomembrane system (H). C, empty-vector control for D–H. Bar = 100 µm (C–F); = 50 µm (G, H).

**Figure 3:**
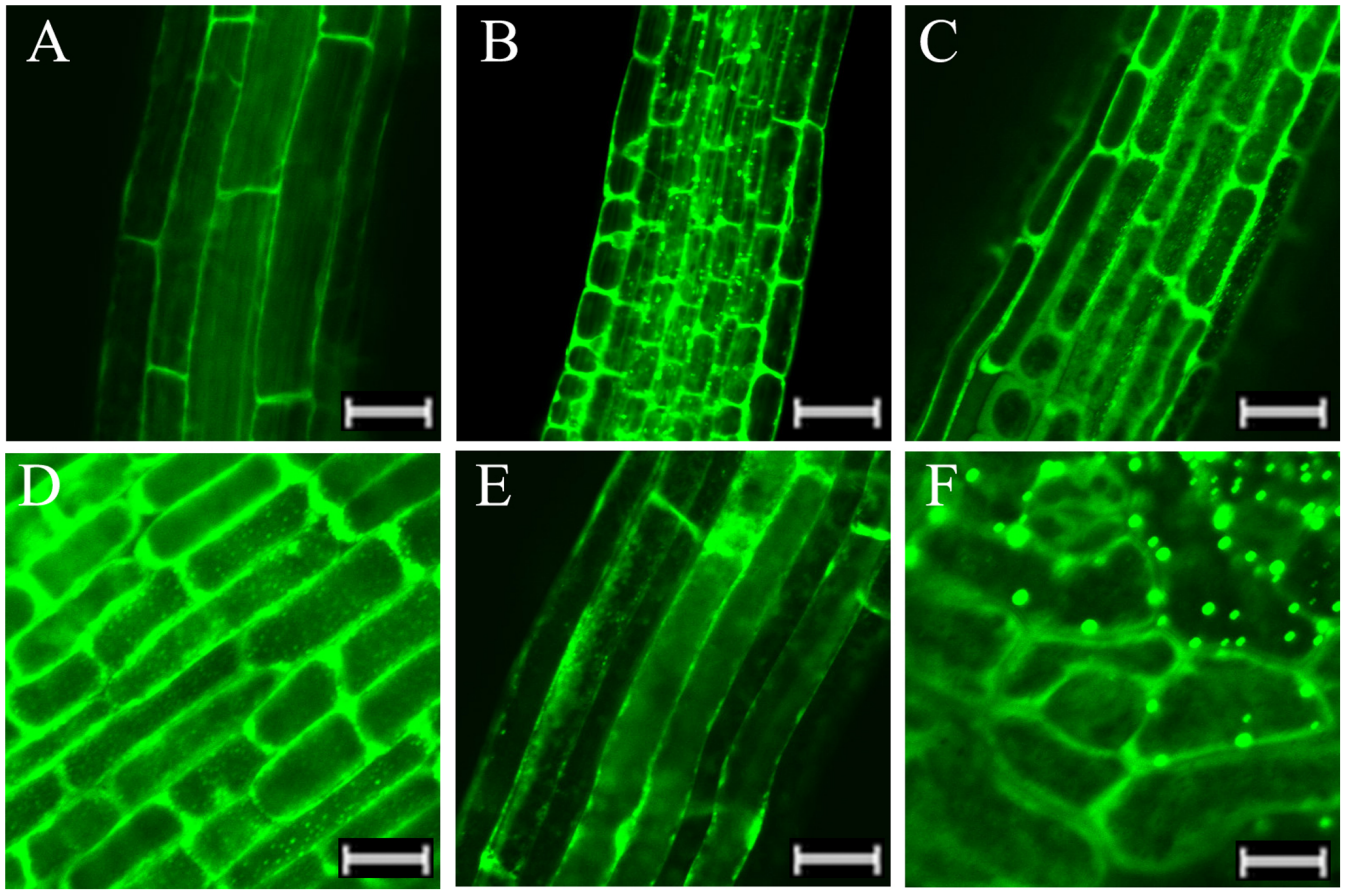
*LimHSP16.45-GFP* expression profiles in abiotic-stressed *Arabidopsis*. Fluorescence intensity was increased over untreated control when seedlings were exposed to 45°C for 1 h (B), 4°C for 4 h (C), 100 mM NaCl for 7 d (D), or 2 mM H_2_O_2_ for 7 d (E). Heat shock granules (HSGs) were formed in roots from overexpressing *Arabidopsis* in response to heat (B) or salinity (D); or in anthers even in absence of abiotic stress (F). Bar = 50 µm (A–C, E, F); = 30 µm (D).

We constructed a vector for the over-expressed *LimHSP16.45-GFP* fusion protein, driven by the 35S promoter (Fig. 2A). After transformation of the construct into *Arabidopsis* and expression of the fusion protein, we obtained four independent transgenic lines for LimHSP16.45-GFP overexpression (OE1, OE2, OE3, and OE4). Expression was higher in OE1 and OE2 compared to OE3 and OE4 (Fig. 2B). LimHSP16.45-GFP was constitutively expressed, with more fluorescence being detected in the apical meristems of roots (Fig. 2D), stems (Fig. 2E), and anthers (Fig. 2F). Intracellularly, LimHSP16.45 was localized to the membrane and endomembrane system (Fig. 2G, H). Expression was greater in the stomatal guard cells than in other epidermic cells of the leaves from transgenic *Arabidopsis* (Fig. 3G), suggesting that LimHSP16.45 maybe has a role in stomatal regulation.

### Expression of LimHSP16.45-GFP is Induced by Abiotic Stress, and Heat Shock Granules (HSGs) are Formed under Heat or Salinity

Expression of Lim-HSP16.45 in David Lily was significantly increased when plants were exposed to either low or high temperatures, high salt, or oxidative stress. When compared with the untreated controls (Fig. 3A), the fluorescence intensity of LimHSP16.45-GFP in transgenic *Arabidopsis* was enhanced when seedlings were treated for 1 h at 45°C or 4 h at 4°C (Fig. 3B, C), or when exposed for 7 d to 100 mM NaCl (Fig. 3D) or 2 mM H_2_O_2_ (Fig. 3E). In addition, HSGs were produced in transgenic roots in response to heat or high salt (Fig. 3B, D), as well as in the anthers even when no stress treatment had been applied (Fig. 3F). We speculated that LimHSP16.45 forms oligomers that have a physiological function under temperature or salinity stress.

### Heterologous Expression of LimHSP16.45-GFP Improves the Viability of *Arabidopsis* Cells under High Temperature Stress or High Salt Stress

**Figure 4:**
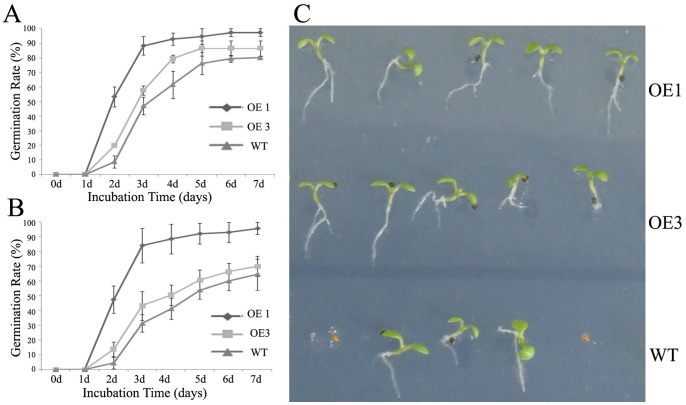
Effects of *LimHSP16.45-GFP* overexpression on tolerance to high temperature stress in *Arabidopsis*. Germination rates were determined for WT and transgenic Lines OE1 and OE3. Prior to germination, seeds were heat-shocked (45°C) for 1 h (A) or 2 h (B). Rates were calculated for 1 to 7 d afterward. C, Seven-day-old seedlings of OE1, OE3, and WT after 1 h of heat stress.

In David Lily, LimHSP16.45 is highly expressed during the late zygotene to pachytene stages of meiotic prophase I in pollen mother cells and tapetal cells of *David Lily*, and its expression can also be induced by heat or cold [16]. To evaluate its possible *in vivo* functioning in heat-stressed transgenic *Arabidopsis*, we monitored seed germination for the WT and transgenic Lines OE 1 and OE 3, which over-expressed LimHSP16.45-GFP. Seeds were treated at 45°C for 1 h (Fig. 4A, C) or 2 h (Fig. 4B) prior to germination, and observations were recorded for the next 1 to 7 d. Not only did typical HSGs form (Fig. 3B), but also the germination rate rose in response to this heat stimulus. These data demonstrated that the transgenic lines had greater cell viability compared with the control, suggesting that overexpression of LimHSP16.45 has an important influence on the plant response to high temperature stress.

**Figure 5:**
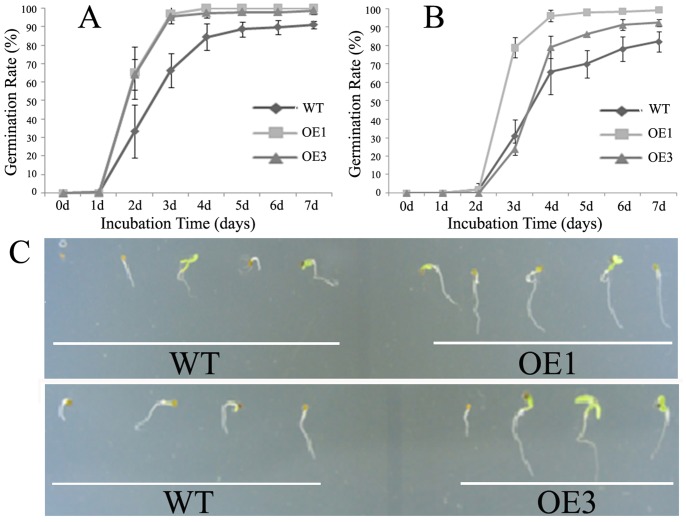
Effects of *LimHSP16.45-GFP* overexpression on tolerance to high salt stress in *Arabidopsis*. Germination rates were calculated for WT and transgenic Lines OE1 and OE3 after 1–7 d exposure to 100 mM NaCl (A) or 150 mM NaCl (B). C, Ten-day-old seedlings of OE1, OE3, and WT growing in MS media containing 100 mM NaCl.

Expression of LimHSP16.45-GFP was stimulated by treatment with 100 mM NaCl (Fig. 1, 3D), and HSGs formed after 7 d of stress (Fig. 3D). Therefore, we examined germination rates in transgenic lines OE1 and OE3 and in the WT after 1 to 7 d of treatment with 100 mM NaCl (Fig. 5A, C) or 150 mM NaCl (Fig. 5B). Under this stress, LimHSP16.45 improved germination, implying that it has a physiological function in response to salinity.

### Overexpression of LimHSP16.45-GFP Induces more SOD and CAT Activity in Transgenic Plants than in the WT

**Figure 6:**
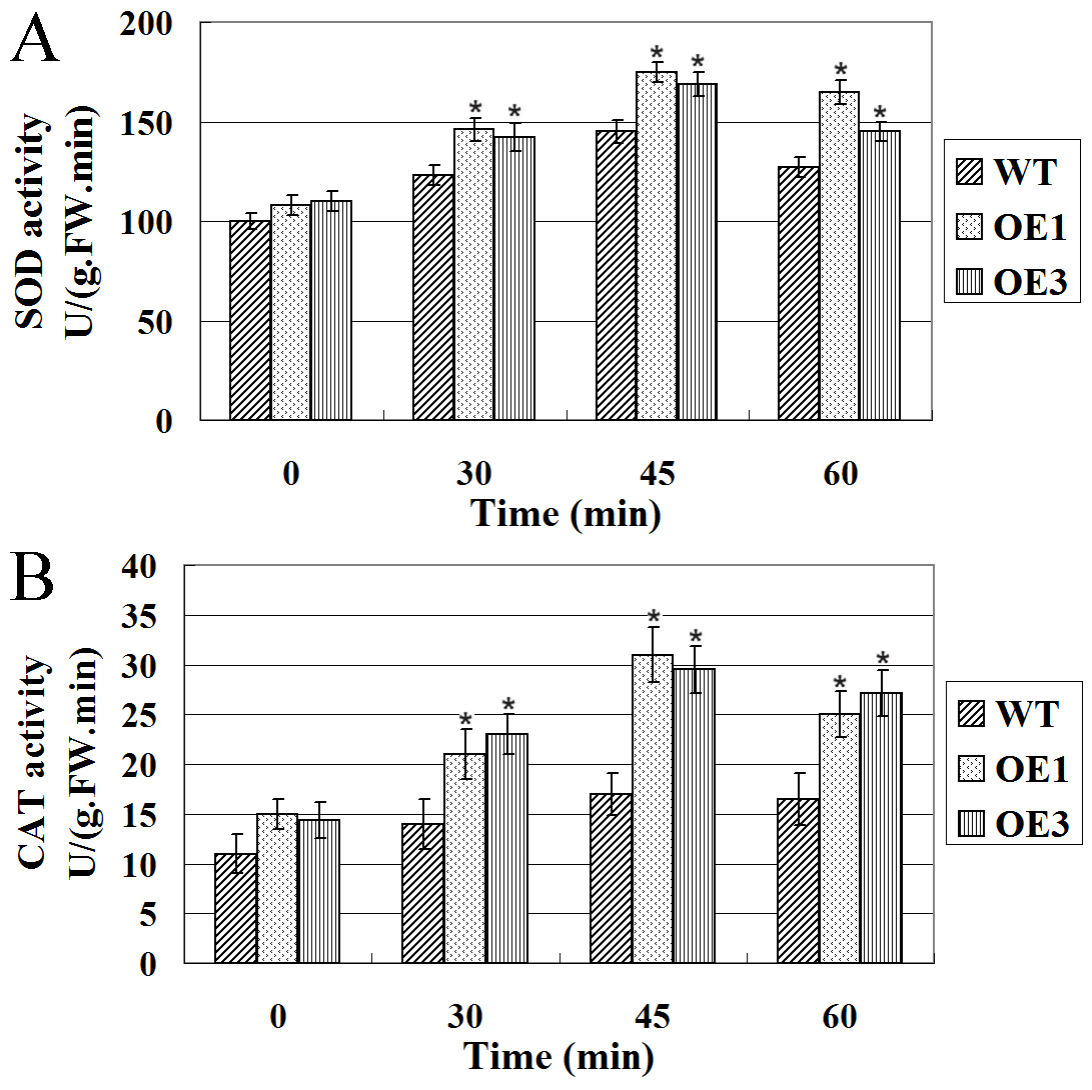
Enzyme activities in response to high temperature stress. Activities of SOD (A) and CAT (B) were higher in Lines OE1 and OE3 than in WT, with or without stress application. *Indicates significant groups, compared with the control (p<0.05).

In plants, various abiotic stresses lead to the overproduction of reactive oxygen species. These toxic ROS can damage proteins, lipids, carbohydrates, and DNA, ultimately resulting in oxidative stress. As part of their antioxidant machinery, plants possess a very efficient system of enzymes that work in concert to control the cascades of uncontrolled oxidation, scavenge for ROS, and protect cells from oxidative damage. Here, we monitored enzymatic activities and found that SOD and CAT levels were higher in the transgenic lines than in the WT aftetr high temperature stress (Fig. 6A, 6B). So, we thought that there are some kinds of relationships between overexpression of LimHSP16.45 and stimulation of the activity of ROS-scavenging enzymes. Although APX activity was also detected, no significant differences in its level were noted between the transgenics and WT (data not shown).

### Heterologous Expression of LimHSP16.45-GFP Enhances the Viability of *Arabidopsis* Cells under Oxidative Stress

**Figure 7:**
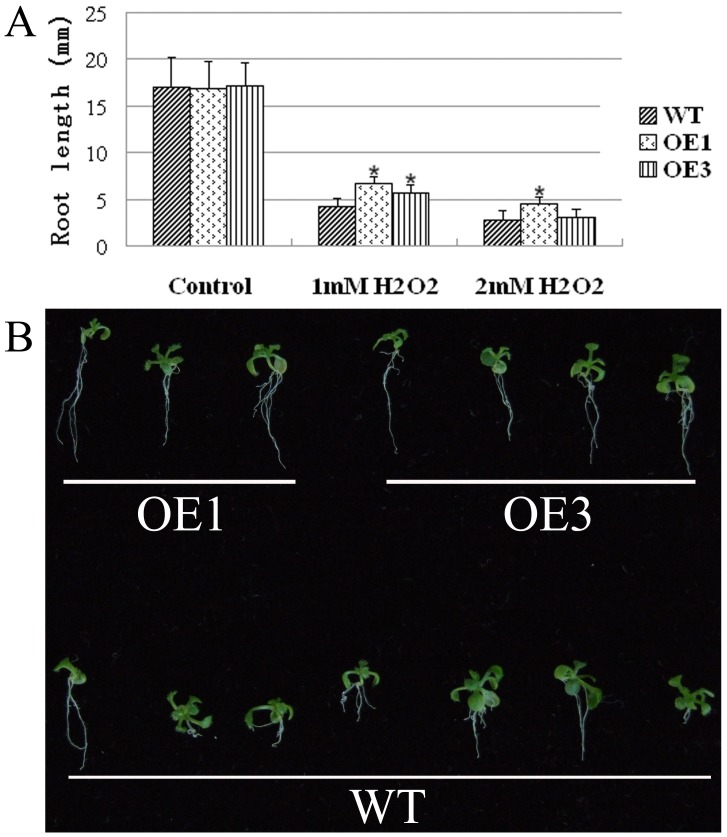
Effects of *LimHSP16.45-GFP* overexpression on tolerance to oxidative stress in *Arabidopsis*. A, Main roots from WT and Lines OE1 and OE3 were measured after exposure for 14 d to 1_2_O_2_. B, Fourteen-day-old seedlings of OE1, OE3, and WT growing in MS media containing 2 mM H_2_O_2_. *Indicates significant groups, compared with the control (p<0.05).

Although expression of LimHSP16.45-GFP was stimulated by treatment with 1 mM H_2_O_2_ (Fig. 1, 3E), oxidative stress did not prompt the formation of HSGs. After 14 d of exposure to 1 mM or 2 mM H_2_O_2_, germination rates did not differ significantly among lines OE1 and OE3 and the WT. However, the roots of transgenic seedlings were significantly longer than those of the WT (Fig. 7A, B). This demonstrated that heterologous expression of LimHSP16.45 can increase seed viability and enhance *Arabidopsis* tolerance to oxidative stress.

## Discussion

### The Localization and Expression Pattern for LimHSP16.45 Indicate its Function in Plants against Abiotic Stresses

LimHSP16.45-GFP is heterologously expressed in the cell membrane and endomembrane system of transgenic *Arabidopsis*. Its amino acid sequence shares very high identity (75.22%) with other plant smHSPs, with the greatest level of homology occurring in the ACD domain [16]. As a molecule chaperone, this domain assists in stabilizing unfolding proteins because of its propensity to associate with denaturing proteins [33]–[34]. Therefore, we speculated that LimHSP16.45, accumulating in the membrane structure, could help avoid ruptures caused there by protein denaturation. LimHSP16.45-GFP was constitutively expressed in the leaves of transgenic *Arabidopsis*, with transcripts being more abundant in the stomatal guard cells than in other epidermic cells. Zou et al. [35] have reported that HSP1 interacts with CPK10 and regulates ABA- and Ca^2+^-mediated stomatal movements in response to drought. Our findings of strong expression in the guard cells suggest that LimHSP16.45 could also be involved in regulating stomatal movements when water is limiting. Thus, expression of smHSPs might be induced by heat, cold, and other abiotic stresses, as also demonstrated by our results with LimHSP16.45 in stressed plants of David Lily and transgenic *Arabidopsis*. All of these data provide evidence that this protein has an important role in plant long-term tolerance to abiotic stresses. In this work, we used 35S promoter for construction of LimHSP16.45-GFP, so induction of LimHSP16.45-GFP in transgenic *Arabidopsis* by abiotic stresses could be a consequence of effect on post-transcriptional and/or post-translational effects.

### LimHSP16.45 is Recruited to Form HSGs under High Temperature or Salinity Stresses

In our transgenic *Arabidopsis*, HSGs formed in response to high temperature and salt exposure. These HSGs are aggregates of electron-dense cytoplasmic particles that contain smHSPs and HSP70 families, and they are found in almost all tissues and plant species [36]–[39]. This formation follows a specific assembly process, in which HSP CII is a prerequisite for stress-induced auto-aggregation and the recruitment of smHSP into HSGs [40]. In plants, most smHSPs are oligomers; our fluorescence observations of HSGs under stress were in accord with theoretical predictions. Therefore, we could conclude that LimHSP16.45 combines with the membrane as a monomer under normal conditions. However, as seen with our temperature- or salt-stressed transgenic *Arabidopsis*, this protein is then released to the cytoplasm to form oligomers that bind to substrate proteins as molecular chaperones. In addition, we observed HSG formation in the anthers of transgenic *Arabidopsis* even in the absence of any abiotic stress. We had previously shown with David Lily that LimHSP16.45 protects the pollen mother cells and tapetal cells against damage from extreme temperatures [16]. Therefore, this protein likely has an important function during specific stages of anther development.

### LimHSP16.45 Overexpression Enhances Cell Viability in *Arabidopsis* under Abiotic Stresses

Because plants are sessile organisms, they cannot escape unfavorable environmental situations. Therefore, their accumulation of smHSPs may indicate a special role to ensure survival under adverse conditions and during their post-stress recovery period [12]. In *vivo*, these smHSPs can act as molecular chaperones to bind partially denatured proteins, thus preventing irreversible protein inactivation and aggregation, and subsequently enhancing thermotolerance [33], [41]. Our earlier data demonstrated that LimHSP16.45 has molecular chaperone activity for luciferase, and can help in luciferase refolding after heat-shock treatment [16].

In the current study, we found that LimHSP16.45-GFP overexpression was induced by various abiotic stresses. This led us to investigate further how its heterologous expression affects plant stress tolerance. Here, the germination rate for *Arabidopsis* seed was increase upon exposure to high temperature or salinity. Moreover, the roots of transgenic lines were longer than those of the WT following H_2_O_2_ treatment. These data demonstrated that our transgenic lines had greater cell viability compared with the control under high temperature, salinity, or oxidative stress. We have already reported that ectopic expression of *LimHSP16.45* enhances the viability of *Escherichia coli* cells exposed to high or low temperatures, and that it can guard pollen mother cells and tapetal cells against extreme temperatures in David Lily [16]. Therefore, we conclude that LimHSP16.45 protects cells against harsh environmental conditions during many life stages.

### LimHSP16.45 Enhances Plant Tolerances to Abiotic Stresses by Stimulating the Activity of ROS-Scavenging Enzymes

In plants, various abiotic stresses lead to ROS overproduction, which can damage proteins, lipids, carbohydrates, and DNA [42]. Superoxide dismutase is the most effective intracellular enzymatic antioxidant. Ubiquitous in all aerobic organisms and in all sub-cellular compartments prone to ROS-mediated oxidative stress, SOD provides the first line of defense against the toxic effects of elevated ROS levels [42]. Catalases are tetrameric heme-containing enzymes with the potential to dismutate H_2_O_2_ directly into H_2_O and O_2_. These CATs are indispensable for ROS-detoxification during stressful periods [43]–[44]. Finally, APX is thought to have the most essential role in scavenging ROS and protecting cells in higher plants [45]–[46]. Here, in the presence of heat, SOD and CAT activities were higher in the transgenic lines than in the WT. Thus, overexpression of ROS-scavenging enzymes, e.g., isoforms of SOD and CAT, enhanced tolerance to abiotic stresses in transgenic *Arabidopsis* because of its efficient capacity for ROS-scavenging. Thus, model species that can scavenge and/or control the levels of cellular ROS will be useful in future efforts to develop plants that can withstand harsh environmental conditions.

In summary, we showed here that heterologous expression of LimHSP16.45-GFP protects plants against high temperature, high salt, and oxidative stresses. This is accomplished in two ways. First, LimHSP16.45 prevents irreversible protein aggregation and maintains denatured proteins, working as a molecular chaperone. Second, abiotic stress tolerance is enhanced through the overexpression of ROS-scavenging enzymes such as SOD and CAT. Because of its strong expression in the guard cells, we believe that LimHSP16.45 probably can regulate stomatal movement during times of drought. Sato and Yokoya [6] have already shown that overexpression of a smHSP can improve drought tolerance in rice. Therefore, future studies should investigate whether overexpression of LimHSP16.45 can enhance the tolerance of David Lily against various abiotic stresses.

## References

[1] Vierling E (1991) The roles of heat shock proteins in plants. Plant Mol Biol 42: 579–620.

[2] Cabane M, Calvet P, Vincens P, Boudet AM (1993) Characterization of chilling-acclimation-related proteins in soybean and identification of one as a member of the heat shock protein (HSP70) family. Planta 190: 346–353. 7763662 10.1007/BF00196963

[3] Krishna P, Sacco M, Cherutti JF, Hill S (1995) Cold-induced accumulation of hsp90 transcripts in Brassica napus. Plant Physiol 107: 915–923. 12228411 10.1104/pp.107.3.915 PMC157208

[4] Waters ER, Lee GJ, Vierling E (1996) Evolution, structure and function of the small heat shock proteins in plants. J Exp Bot 47: 325–338.

[5] Keeler SJ, Boettger CM, Haynes JG, Kuches KA, Johnson MM, et al (2000) Acquired thermotolerance and expression of the HSP100/ClpB genes of Lima Bean. Plant Physiol 123: 1121–1132. 10889261 10.1104/pp.123.3.1121 PMC59075

[6] Sato Y, Yokoya S (2008) Enhanced tolerance to drought stress in transgenic rice plants overexpressing a small heat-shock protein smHSPs17.7. Plant Cell Reports 27: 329–334. 17968552 10.1007/s00299-007-0470-0

[7] Parsell DA, Lindquist S (1993) The function of heat-shock proteins in stress tolerance: degradation and reactivation of proteins. Annu Rev Genet 27: 437–96. 8122909 10.1146/annurev.ge.27.120193.002253

[8] Scharf KD, Siddique M, Vierling E (2001) The expanding family of Arabidopsis thaliana small heat stress proteins and a new family of proteins containing alpha-crystallin domains (Acd proteins). Cell Stress Chaperones 6: 225–237. 11599564 10.1379/1466-1268(2001)006<0225:tefoat>2.0.co;2 PMC434404

[9] Helm KW, LaFayette PR, Nagao RT, Key JL, Vierling E (1993) Localization of small heat shock proteins to the higher plant endomembrane system. Mol Cell Biol 13: 238–247. 8417329 10.1128/mcb.13.1.238-247.1993 PMC358903

[10] Osteryoung KW, Vierling E (1994) Dynamics of small heat shock protein distribution within the chloroplasts of higher plants. J Biol Chem 269: 28676–28682. 7961818

[11] Lee GJ, Roseman AM, Saibil HR, Vierling E (1997) A small heat shock protein stably binds heat-denatured model substrates and can maintain a substrate in a folding-competent state. EMBO J 16: 659–671. 9034347 10.1093/emboj/16.3.659 PMC1169668

[12] Low D, Brandle K, Nover L, Forreiter C (2000) Cytosolic heat-stress proteins Hsp17.7 class I and Hsp17.3 class II of tomato act as molecular chaperones in vivo. Planta 211: 575–582. 11030557 10.1007/s004250000315

[13] Sun W, Bernard C, van de Cotte B, van Montagu M, Verbruggen N (2001) At-HSP17.6A, encoding a small heat-shock protein in Arabidopsis, can enhance osmotolerance upon overexpression. The Plant Journal 27: 407–415. 11576425 10.1046/j.1365-313x.2001.01107.x

[14] Basha E, Friedrich KL, Vierling E (2006) The N-terminal arm of small heat shock proteins is important for both chaperone activity and substrate specificity. J Biol Chem 281: 39943–39952. 17090542 10.1074/jbc.M607677200

[15] Ahrman E, Lambert W, Aquilina JA, Robinson CV, Emanuelsson CS (2007) Chemical cross-linking of the chloroplast localized small heat-shock protein, Hsp21, and the model substrate citrate synthase. Protein Sci 16: 1464–1478. 17567739 10.1110/ps.072831607 PMC2206695

[16] Mu C, Wang S, Zhang S, Pan J, Chen N, et al (2011) Small heat shock protein LimHSP16.45 protects pollen mother cells and tapetal cells against extreme temperatures during late zygotene to pachytene stages of meiotic prophase I in David Lily. Plant Cell Reports 30: 1981–1989. 21678060 10.1007/s00299-011-1106-y

[17] Neta-Sharir I, Isaacson T, Lurie S, Weiss D (2005) Dual role for tomato heat shock protein 21: protecting photosystem II from oxidative stress and promoting color changes during fruit maturation. Plant Cell 17: 1829–1838. 15879560 10.1105/tpc.105.031914 PMC1143080

[18] Volkov RA, Panchuk II, Schoffl F (2005) Small heat shock proteins are differentially regulated during pollen development and following heat stress in tobacco. Plant Mol Biol 57(4): 487–502. 15821976 10.1007/s11103-005-0339-y

[19] Zou J, Liu AL, Chen XB, Zhou XY, Gao GF, et al (2009) Expression analysis of nine rice heat shock protein genes under abiotic stresses and ABA treatment. J Plant Physiol 166(8): 851–861. 19135278 10.1016/j.jplph.2008.11.007

[20] Prandl R, Kloske E, SchOffl F (1995) Developmental regulation and tissue-specific differences of heat shock gene expression in transgenic tobacco and Arabidopsis plants. Plant Mol Biol 28: 73–82. 7787189 10.1007/BF00042039

[21] Malik MK, Slovin JP, Hwang CH, Zimmerman JL (1999) Modified expression of a carrot small heat shock protein gene, hsp17.7, results in increased or decreased thermotolerance double danger. The Plant Journal 20: 89–99. 10571868 10.1046/j.1365-313x.1999.00581.x

[22] Hamilton EW, Heckathorn SA (2001) Mitochondrial adaptations to NaCl. Complex I is protected by anti-oxidants and small heat shock proteins, whereas complex II is protected by proline and betaine. Plant Physiology 126: 1266–1274. 11457977 10.1104/pp.126.3.1266 PMC116483

[23] Lee BH, Won SH, Lee HS, Miyao M, Chung WI, et al (2000) Expression of the chloroplast-localized small heat shock protein by oxidative stress in rice. Gene 245: 283–290. 10717479 10.1016/s0378-1119(00)00043-3

[24] Perez DE, Hoyer JS, Johnson AI, Moody ZR, Lopez J, et al (2009) BOBBER1 is a noncanonical Arabidopsis small heat shock protein required for both development and thermotolerance. Plant Physiol 151: 241–252. 19571304 10.1104/pp.109.142125 PMC2735987

[25] Sanmiya K, Suzuki K, Egawa Y, Shono M (2004) Mitochondrial small heat-shock protein enhances thermotolerance in tobacco plants. FEBS Lett 557: 65–268. 10.1016/s0014-5793(03)01494-7 14741379

[26] Prieto-Dapena P, Castano R, Almoguera C, Jordano J (2006) Improved resistance to controlled deterioration in transgenic seeds. Plant Physiol 142: 1102–1112. 16998084 10.1104/pp.106.087817 PMC1630740

[27] Guo SJ, Zhou HY, Zhang XS, Li XG, Meng QW (2007) Overexpression of CaHSP26 in transgenic tobacco alleviates photoinhibition of PSII and PSI during chilling stress under low irradiance. J Plant Physiol 164: 126–136. 16513207 10.1016/j.jplph.2006.01.004

[28] Mu C, Wang S, Pan J, Zhang S, Yu G, et al (2012) Analysis of Highly Expressed Genes in the Late Zygotene to Pachytene Stages of Meiotic Prophase I in David Lily. Russian Journal of Plant Physiology 59(3): 389–397.

[29] Clough SJ, Bent AF (1998) Floral dip: A simplified method for Agrobacterium-mediated transformation of Arabidopsis thaliana. Plant J 16: 735–743. 10069079 10.1046/j.1365-313x.1998.00343.x

[30] Murashige T, Skoog F (1962) A revised medium for rapid growth and bioassays with tobacco tissue cultures. Physiol Plant 15: 473–497.

[31] Beyer WF Jr, Fridovich I (1987) Assaying for superoxide dismutase activity: some large consequences of minor changes in conditions. Anal Biochem 161(2): 559–566. 3034103 10.1016/0003-2697(87)90489-1

[32] Beaumont F, Jouve HM, Gagnon J, Gaillard J, Pelmont J (1990) Purification and properties of a catalase from potato tubers (*Solanum tuberosum*). Plant Sci 72: 19–26.

[33] Horwitz J (1992) α-Crystallin can function as a molecular chaperone. Proc Natl Acad Sci USA 89: 10449–10453. 1438232 10.1073/pnas.89.21.10449 PMC50356

[34] Merck KB, Groenen PJ, Voorter CE, de Haard-Hoekman WA, Horwitz J, et al (1993) Structural and functional similarities of bovine α-crystallin and mouse small heat-shock protein. J Biol Chem 268: 1046–52. 8093449

[35] Zou JJ, Wei FJ, Wang C, Wu JJ, Ratnasekera D, et al (2010) Arabidopsis Calcium-Dependent Protein Kinase CPK10 Functions in Abscisic Acid- and Ca2+-Mediated Stomatal Regulation in Response to Drought Stress. Plant Physiology 154: 1232–1243. 20805328 10.1104/pp.110.157545 PMC2971602

[36] Nover L, Scharf KD, Neumann D (1983) Formation of cytoplasmic heat shock granules in tomato cell cultures and leaves. Mol. Cell Biol. 3: 1648–1655. 10.1128/mcb.3.9.1648-1655.1983 PMC370018 6633535

[37] Nover L, Scharf KD, Neumann D (1989) Cytoplasmic heat shock granules are formed from precursor particles and are associated with a specific set of mRNAs. Mol. Cell. Biol. 9: 1298–1308. 10.1128/mcb.9.3.1298-1308.1989 PMC362722 2725500

[38] Smykal P, Hrdy I, Pechan PM (2000) High-molecular-mass complexes formed in vivo contains smHSPs and HSP70 and display chaperone-like activity. Eur. J. Biochem. 267: 2195–2207. 10.1046/j.1432-1327.2000.01223.x 10759842

[39] Weber C, Nover L, Fauth M (2008) Plant stress granules and mRNA processing bodies are distinct from heat stress granules. The Plant Journal 56: 517–530. 18643965 10.1111/j.1365-313X.2008.03623.x

[40] Kirschner M, Winkelhaus S, Thierfelder JM, Nover L (2000) Transient expression and heat-stress-induced co-aggregation of endogenous and heterologous small heat-stress proteins in tobacco protoplasts. Plant J. 24: 397–412. 10.1046/j.1365-313x.2000.00887.x 11069712

[41] Jakob U, Gaestal M, Engel K, Buchner J (1993) Small heat shock proteins are molecular chaperons. J. Biol Chem. 268: 1517–1520. 8093612

[42] Gill SS, Tuteja N (2010) Reactive oxygen species and antioxidant machinery in abiotic stress tolerance in crop plants. Plant Physiology and Biochemistry 48: 909–930. 20870416 10.1016/j.plaphy.2010.08.016

[43] Garg N, Manchanda G (2009) ROS generation in plants: boon or bane? Plant Biosys. 143: 88–96.

[44] Azpilicueta CE, Benavides MP, Tomaro ML, Gallego SM (2007) Mechanism of CATA3 induction by cadmium in sunflower leaves. Plant Physiol. Biochem. 45: 589–595. 10.1016/j.plaphy.2007.04.005 17583519

[45] Mobin M, Khan NA (2007) Photosynthetic activity, pigment composition and antioxidative response of two mustard (Brassica juncea) cultivars differing in photosynthetic capacity subjected to cadmium stress. J. Plant Physiol. 164: 601–610. 10.1016/j.jplph.2006.03.003 16621132

[46] Singh S, Khan NA, Nazar R, Anjum NA (2008) Photosynthetic traits and activities of antioxidant enzymes in blackgram (Vigna mungo L. Hepper) under cadmium stress. Am. J. Plant Physiol. 3: 25–32.

